# Distribution of the Octopamine Receptor AmOA1 in the Honey Bee Brain

**DOI:** 10.1371/journal.pone.0014536

**Published:** 2011-01-18

**Authors:** Irina Sinakevitch, Julie A. Mustard, Brian H. Smith

**Affiliations:** Arizona State University, School of Life Sciences, Tempe, Arizona, United States of America; Freie Universitaet Berlin, Germany

## Abstract

Octopamine plays an important role in many behaviors in invertebrates. It acts via binding to G protein coupled receptors located on the plasma membrane of responsive cells. Several distinct subtypes of octopamine receptors have been found in invertebrates, yet little is known about the expression pattern of these different receptor subtypes and how each subtype may contribute to different behaviors. One honey bee (*Apis mellifera*) octopamine receptor, AmOA1, was recently cloned and characterized. Here we continue to characterize the AmOA1 receptor by investigating its distribution in the honey bee brain. We used two independent antibodies produced against two distinct peptides in the carboxyl-terminus to study the distribution of the AmOA1 receptor in the honey bee brain. We found that both anti-AmOA1 antibodies revealed labeling of cell body clusters throughout the brain and within the following brain neuropils: the antennal lobes; the calyces, pedunculus, vertical (alpha, gamma) and medial (beta) lobes of the mushroom body; the optic lobes; the subesophageal ganglion; and the central complex. Double immunofluorescence staining using anti-GABA and anti-AmOA1 receptor antibodies revealed that a population of inhibitory GABAergic local interneurons in the antennal lobes express the AmOA1 receptor in the cell bodies, axons and their endings in the glomeruli. In the mushroom bodies, AmOA1 receptors are expressed in a subpopulation of inhibitory GABAergic feedback neurons that ends in the visual (outer half of basal ring and collar regions) and olfactory (lip and inner basal ring region) calyx neuropils, as well as in the collar and lip zones of the vertical and medial lobes. The data suggest that one effect of octopamine via AmOA1 in the antennal lobe and mushroom body is to modulate inhibitory neurons.

## Introduction

The biogenic amine octopamine acts as a neurotransmitter, neuromodulator and neurohormone in the nervous system of invertebrates [1], [2]. Numerous functions have been assigned to octopamine including regulation of lipid versus carbohydrate metabolism in insect flight muscle [3], [4], increasing levels of arousal [5]–[7], modulation of sensory perception [8]–[12], aggression [13], [14], control of locomotion [15]–[17] and signaling the presence of reward in appetitive olfactory learning [18]–[21]. In the honey bee, octopamine has also been linked with social behaviors such as nestmate recognition [22], hygienic behavior [23] and division of labor [24], [25]. In order to understand the specificity of the action of octopamine in so many different types of behaviors, it will be necessary to describe not only where octopamine is released but also the distribution of different octopamine receptor subtypes in the brain.

Several studies have investigated the sites of octopamine release in the insect central and peripheral nervous system [26]–[33]. In the honey bee brain, different clusters of octopaminergic neurons release octopamine in distributed areas of the brain. For example, many areas receive input from the octopaminergic ventral unpaired median neurons (central VUM neurons) identified in part by cell bodies that lie on the median (midline) ventral part of the subesophageal ganglion [30], [32], [34], [35]. Two of these neurons, VUMmx1 and VUMmd1, have a primary neurite that projects through the midline tract of the subesophageal ganglion and gives rise to two symmetrical secondary axons that project collaterals to the antennal lobes, lateral horn, lateral protocerebrum and to the lip and basal ring of the mushroom body calyces [34], [35]. Collectively, these neuropils process many of the different types of sensory stimuli that honey bees are capable of associating with reinforcement.

Octopamine also plays an important role in modulating activity in the visual neuropil and the central complex of the honey bee brain. There is a group of six neurons, group G3a [32], located in the medial part of the tritocerebrum that send their axons through the posterior optic tract to provide extensive arborizations in the lobula, medulla and lamina of the compound eyes. Some of these neurons send their collaterals into the optic lobe, the ocelli and central complex. Octopamine application in the optic lobes enhances directional sensitivity of the antennal reflex to visual stimuli [8], and it has modulatory effects on motion-sensitive neurons in the lobula [36]. In addition, the central complex receives octopaminergic innervation from neurons located in the posterior of the brain. These neurons connect the protocerebral bridge with the ellipsoid body and lateral protocerebrum [32].

Octopamine acts via binding to different G protein coupled receptors that regulate intracellular levels of cyclic AMP (cAMP) or calcium [37], [38]. Four distinct octopamine receptor subtypes have been cloned and characterized from *Drosophila* [39]–[41], and homologs of these receptors are found in the honey bee genome sequence [38], [42]. The characterized fruit fly octopamine receptors and their putative honey bee orthologs cluster into two classes [38]. One class contains OAMB, and receptors in this group probably act to regulate intracellular calcium levels [39], [43]. The other octopamine receptors are closely related to each other and cluster together in a second class. Octopamine receptors in the second group act through the cAMP second messenger pathway, as stimulation with octopamine results in an increase in cAMP [38].

To date, only one octopamine receptor (AmOA1) has been cloned and characterized from honey bee [43]. When expressed in HEK cells, activation of AmOA1 receptors by octopamine leads to oscillations of intracellular Ca^2+^ levels and a relatively small increase in cAMP levels [43]. AmOA1 is the ortholog of the fruit fly OAMB (also known as DmOA1A or DmOctα) receptor [40], [41]. Although OAMB was originally believed to regulate cAMP, recent evidence suggests that, as for AmOA1, activation of this receptor leads to increases in Ca^2+^ [39]. Down regulation of the expression of AmOA1 via RNA interference significantly reduces olfactory learning [18], suggesting that AmOA1 receptors are an important part of the OA reinforcement pathway.

Previous work on the distribution of *Amoa1* transcript in the honey bee brain suggests that this biogenic amine receptor is widely expressed in many somata throughout the brain [43]. While these studies have provided a wealth of important information, using antibodies against the AmOA1 receptor for immunolabeling can reveal more about the exact locations of the receptor protein thereby suggesting a role for a receptor in specific neuroanatomical pathways. In the present study, we use two polyclonal antibodies generated (in rabbit and goat) against two different peptide sequences in the C-terminus of AmOA1 to examine the distribution of the AmOA1 receptor in the honey bee brain. We describe AmOA1 immunolabeling specifically in the antennal lobes, mushroom body, central complex, optic lobes and subesophageal neuropils of forager honey bees. Furthermore, double immunofluorescence staining using anti-GABA and anti-AmOA1 receptor antibodies revealed that the receptor is expressed in the GABAergic local interneurons in the antennal lobe and in the GABAergic feedback neurons in the mushroom body. Thus we present here for the first time evidence that the AmOA1 receptor is expressed in the inhibitory pathways in the olfactory learning and memory neuropils of the bee brain.

## Materials and Methods

### Animals

Honey bees (*Apis mellifera*) used in this study were adult New World Carniolan pollen foragers from colonies maintained at Arizona State University. We used fruit fly stocks to analyze specificity of our antibodies. Fly stocks and crosses were maintained at 25°C on standard corn meal-yeast-agar medium. The following strains were used: wild-type Canton-S; *oamb^96^* mutant with a deletion in the OAMB locus [44] kindly provided by Dr. B. Dickson (Institute of Molecular Pathology, Vienna, Austria).

### Primary antisera

Rabbit polyclonal antibodies against AmOA1 (Ranti-AmOA1) were generated against a 15 amino acid peptide (NH_2_-DFRFAFKSIICKCFC-OH) conjugated to keyhole limpet hemocyanin (KLH) via glutaraldehyde by Alpha Diagnostic International (San Antonio, TX). AmOA1 antiserum from rabbit was purified by preabsorption with glutaraldehyde treated KLH. The purified antiserum from rabbit 65-4 was used for all the immunocytochemical analyses reported below.

Goat polyclonal antibodies against AmOA1 (Ganti-AmOA1) were generated against a different AmOA1 peptide sequence. In this case the synthetic peptide acetyl-AMRNDRSPSYSMQVPQQGC-amide was used, which corresponds to amino acids 547–564 of AmOA1 (21st Century Biochemicals, Inc., Marlborough, MA). The peptide was analyzed by HPLC and nanospray MS and the sequence confirmed by CID MS/MS (MS CheckTM, 21st Century Biochemicals). The peptide was conjugated to KLH using MBS and dialyzed prior to injection. The fourth bleeds were tested by ELISA and the antibodies were affinity purified using the above peptide covalently attached to cross-linked agarose beads.

Octopamine antiserum was obtained from rabbit immunized with octopamine conjugated to bovine serum albumin via glutaraldehyde (BSA) [45]. Its specificity to octopamine has been described [46]. This octopamine antiserum has been used previously to characterize octopamine-like immunoreactivity in the honey bee brain [32].

GABA antiserum (GEMAC; Talence, France) was raised in rabbit using GABA conjugated with glutaraldehyde to BSA, bovine hemoglobin, or poly-L-lysine. The antiserum specificity has been described elsewhere [47]–[49], and it has already been used to characterize GABA in the honey bee visual system [48].

### Immunohistochemistry

Honey bee brains and fly brains were removed in fixative containing 2.5% paraformaldehyde (EMS), 1.5% glutaraldehyde (EMS) in 0.1 M sodium cacodylate buffer (pH 7.0), with 1% sodium metabisulfite (SMB, Sigma) and were incubated in the same solution overnight at 4°C. Fixation with glutaraldehyde produces a background of blade marks in agarose sections [50], [51]. However, the blade marks produce a regular pattern that is differentiable from the neuropil staining. We found that fixative containing glutaraldehyde worked best for staining tissue sections, probably because the AmOA1 antibody was generated with peptide conjugated to carrier protein via glutaraldehyde.

After fixation, whole brains were incubated for 15 minutes in Tris-SMB buffer (0.05 M Tris-HCl, pH 7.5; 0.45% SMB) containing 0.5% NaBH_4_ to saturate double bonds. After washing (4×10 min) in Tris-SMB buffer, brains were embedded in agarose and cut into 60 µm sections.

### Immunohistochemistry with rabbit primary antibodies: Ranti-AmOA1, anti-octopamine and anti-GABA

Sections were washed (6×10 min) in Tris-SMB buffer with 0.5% of Triton X100 (Tris-SMB-TX), then preincubated with 5% Normal Swine Serum (Dakopatts a/s, Glostrup, Denmark) for one hour. Next, the brain sections were incubated overnight with primary antibodies: Ranti-AmOA1 at 1∶1000, or anti-octopamine or anti-GABA at 1∶100 diluted in Tris -SMB-TX. After washing (6×10 min) in 0.05 M Tris, pH 7.5, 0.5% Triton X100 (Tris -TX), sections were incubated overnight in goat anti-rabbit IgG antibodies conjugated to either Alexa 488 (Molecular Probes, Eugene, OR) or Cy5 (Jackson Laboratories, West Grove). After a final wash in 0.05 M Tris-HCl buffer pH 7.5, sections were embedded in mounting medium and collected on a Zeiss LSM 510 confocal microscope (Zeiss, Oberkochen, Germany).

**Figure 1:**
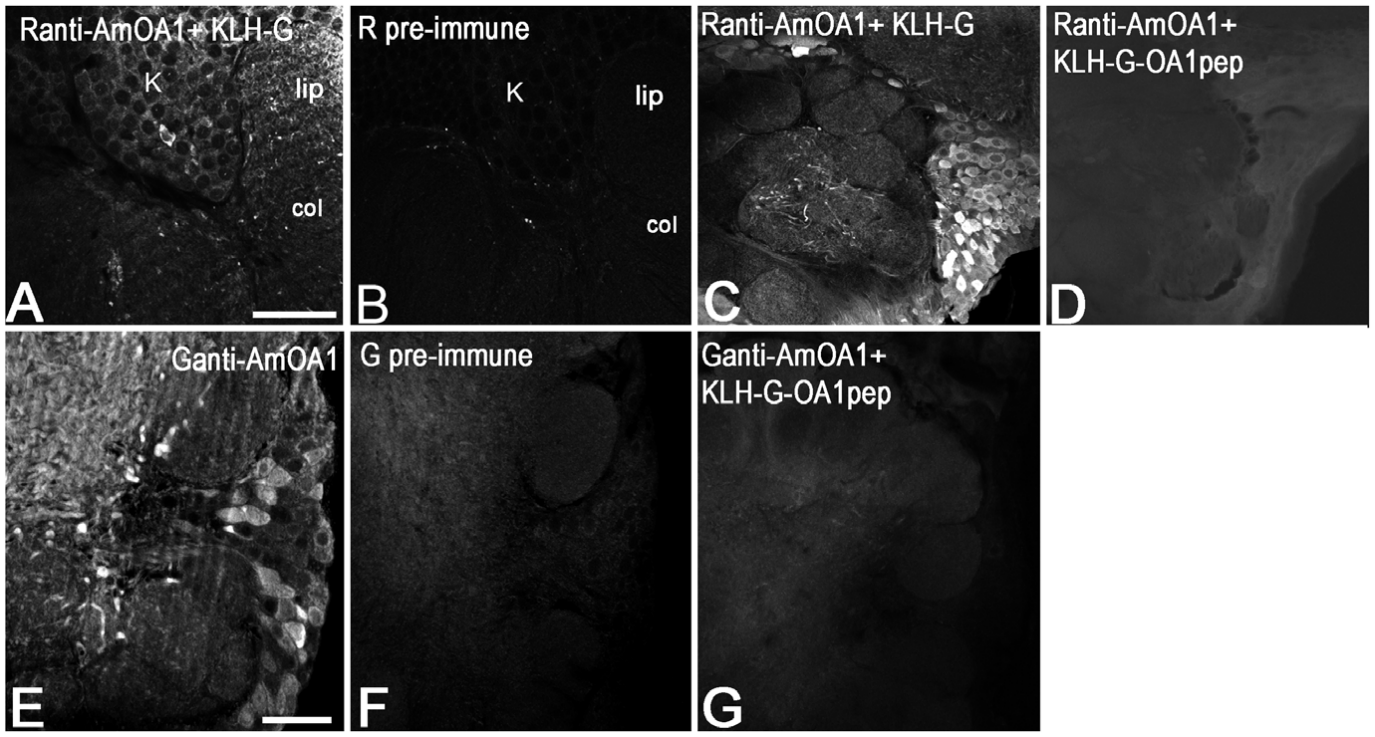
Anti-AmOA1 antibodies are specific for the AmOA1 receptor. Pre- and post-adsorption controls of immunolabeling with anti-AmOA1 antibodies from rabbit (**A–D**) and from goat (**E–G**). Consecutive sections of the mushroom body calyx stained with Ranti-AmOA1 serum (**A**) and pre-immune serum taken from the rabbit before immunization and used at the same dilution as the Ranti-AmOA1 serum (**B**). The Ranti-AmOA1 serum revealed staining in Kenyon cell bodies (K) with different intensity and in processes of the calyx lip and collar (col). **C**: Section of the antennal lobe stained with Ranti-AmOA1 that was preadsorbed with KLH treated with glutaraldehyde (KLH-G) alone. **D**: A section from the same brain stained with Ranti-AmOA1 that was preadsorbed with the AmOA1 peptide conjugated to KLH (KLH-G-OA1pep). **E**: Sections of the antennal lobe stained with affinity purified anti-AmOA1 antibodies from goat (Ganti-AmOA1). **F**: pre-immune serum from the goat before immunization used at the same dilution as the Ganti-AmOA1 serum. **G**: Specific staining from cell bodies and their processes disappeared after pretreatment with the KLH conjugated to the AmOA1 peptide. The confocal images in **D, F** and **G** were adjusted to a higher level of intensity then stained sections in **C, E** to show the image of the antennal lobe. Scale bars: 50 µm.

To examine the specificity of immunostaining with the Ranti-AmOA1 serum, sections were incubated with the secondary antibody in the absence of primary antiserum (not shown), or they were immunoassayed with Ranti-AmOA1 serum (Fig. 1A) or preimmune serum (Fig. 1B). Furthermore, sections were incubated with antibody that had been preadsorbed with glutaraldehyde treated KLH (KLH-G) alone (Fig. 1C) or KLH-G conjugated to the AmOA1 peptide used to make antibodies in rabbit (Fig. 1D). To prepare the peptide conjugates for preadsorbtion, 100 µM of peptide was conjugated to KLH via gluteraldehyde [45].

For double immunofluorescence staining with Ranti-AmOA1 and with anti-GABA, sections labeled with Ranti-AmOA1 were detached from slides and washed in 0.05 M Tris-HCl, pH 7.5. They were then postfixed in 4% paraformaldehyde in PBS with 1% SMB for 20 minutes in order to deactivate the antibodies of the first sequence of staining. Then sections were processed for GABA immunostaining using the same protocol as described above. To compare octopamine (or GABA) immunostaining with anti-AmOA1 staining, we stained alternating sections from the same brain. For control of double immunostaining (Figure S1A) we used Tris-HCl buffer with 5% swine serum instead of GABA antiserum (Fig. S1B) or AmOA1 antiserum (Fig. S1C) on two consecutive sections. In these controls we did not observe any interaction between the reagents of the first and the second sequences of staining.

To test the specificity of GABA immunostaining, working dilutions of GABA antibodies were preincubated overnight with conjugate containing 100 µM hapten conjugated GABA-G-BSA. After preadsorption of the primary antiserum with GABA-G-BSA, the staining was abolished.

### Immunohistochemistry with primary antiserum from goat (Ganti-AmOA1)

After reduction of double bonds with NaBH_4_, sections were washed (6×10 min) in PBS buffer with 0.5% of Triton X100 (PBS-TX), then preincubated with 5% Normal Donkey Serum (Jackson ImmunoResearch) for one hour. The brain sections were then incubated overnight with affinity purified anti-AmOA1antibodies from goat (Ganti-AmOA1) diluted 1∶100 in PBS-TX. After washing (6×10 min) in PBS-TX, sections were incubated overnight in F(ab') _2_ fragments of donkey anti-goat IgG antibodies conjugated to either Cy2 or Cy3 (Jackson Laboratories, West Grove). After a final wash in PBS buffer, sections were embedded in mounting medium and data were collected on a Zeiss LSM 510 confocal microscope (Zeiss, Oberkochen, Germany).

To examine the specificity of the immunostaining with the Ganti-AmOA1 antibodies, sections were incubated with the secondary antibody in the absence of primary antiserum (not shown) or they were immunoassayed with Ganti-AmOA1 (Fig. 1E) or preimmune serum (Fig. 1F). Furthermore, sections were incubated with antibody that had been preadsorbed with KLH conjugated to AmOA1 peptide that was used to make antibodies in goat (Fig. 1G). To prepare the preadsorbed antibody, 400 µM of peptide was conjugated to KLH via glutaraldehyde and incubated with working dilution of Ganti-AmOA1.

For double immunofluorescence staining with Ganti-AmOA1 and GABA, the agarose brain sections were incubated simultaneously with both antibodies, then after a thorough wash in PBS, the secondary antibodies F(ab') _2_ fragments of donkey anti-Goat IgG Cy2 and F(ab') _2_ fragments of donkey anti-Rabbit IgG Cy5 were added in dilution 1∶200 for incubation overnight. For control of staining, both of the secondary antibodies were incubated with sections that had only one of the primary antibodies, and the staining did not show any cross-reaction between the antibodies (Fig. S1D–F).

Procedures with all antibodies were performed at room temperature. Images of sections treated with antibodies preincubated with peptide were taken with the intensity of fluorescence gain equal to or greater than images of antibody treated sections. For comparison, images were collected at the same level of gain and intensity. In Fig. 1D, F, G the image was collected at a higher gain because confocal collection at the same gain as shown in Figures 1C and D resulted in such low intensity that the image was not visible.

## Results

### Specificity of the anti-AmOA1 antibodies

Two different polyclonal antibodies were used in our studies, one raised in rabbit (Ranti-AmOA1) and another raised in goat (Ganti-AmOA1) against two different peptides corresponding to different regions in the cytoplasmic carboxyl terminus of the AmOA1 receptor. Double labeling experiments revealed that these antibodies labeled the same cells and processes in the neuropil of the bee brain (an example of double staining in the antennal lobe is in Figure S1, G1-3). For this reason we do not specify the R or G version of the antibody in the results. Unless otherwise noted in the legends, figures show data from the Ranti-AmOA1 serum.

**Figure 2:**
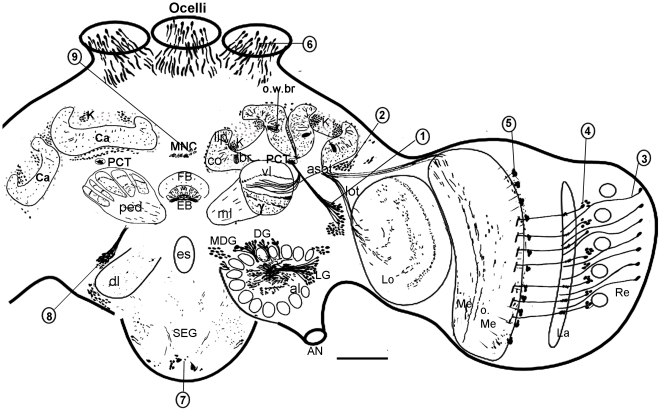
Schematic drawing of AmOA1 immunoreactive neurons and processes in the brain and subesophageal ganglion of the bee made from confocal images of agarose sections of bee brain stained with anti-AmOA1 antibodies. The right hemisphere shows a frontal view of the bee brain with the optic lobe neuropils (Re, retina; La, lamina; Lo, lobula), antennal lobe (al) and summarized mushroom body neuropil; the left hemisphere of the brain shows a more posterior frontal view at the level of the dorsal lobe (dl), pedunculus (ped) and calyces of the mushroom bodies. AN, antennal nerve; DG, dorsal group of antennal lobe neurons; LG, lateral group of antennal lobe neurons; MDG, medial group of antennal lobe neurons. Some neurons that express the AmOA1 receptor could be identified as: 1, PCT (or feedback neuron group); 2, lobula and medulla mushroom body neurons. The cells in this cluster have fibers in the anterior superior optical tract (asot). The arrow shows axons (only the ending of the tract) from these neurons as they enter the medulla serpentine layer. A few AmOA1 positive fibers enter through the lobula optical tract (lot) that connects the lobula and mushroom bodies.; 3, photoreceptor cells in the retina; 4, monopolar cell bodies in the lamina; 5, medulla columnar neurons; 6, photoreceptor cells in the ocelli; 7, a median group of subesophageal intrinsic neurons; 8, ellipsoid body neurons, 9, a group of median neurosecretory cells (MNC) in the pars intercerebralis. Abbreviations: K, Kenyon cell bodies; Ca, calyx of the mushroom bodies; o.w.br, the outer wedge of the basal ring; co, collar; br, basal ring; ped, pedunculus; vl, vertical lobe of the mushroom bodies; ml, medial lobe of the mushroom bodies; γ, gamma lobe of the mushroom bodies; PCT, protocerebral-calycal tract; EB, ellipsoid body; FB, fanshaped body; o.Me, outer medulla; i. Me, inner Medulla; es, esophagus; SEG, subesophageal ganglion. Scale bar: 250 µm.

Both antibodies labeled cell body clusters and many processes in the antennal lobe, mushroom body (calyces, pedunculus, alpha, beta and gamma lobes), optic lobe, subesophageal ganglion and central complex (Fig. 1A, C, E, 2). The immunostained cell body clusters and processes were repeatable from one animal to the next when we compared brains of forager bees caught at the entrance of the same colony. Staining in cell bodies and processes ranged from high to low intensity (Fig. 1A,C,E). This variation is consistent with data from in situ hybridization where differences in gene expression in cell bodies were previously reported [43]. Preadsorption of either antibody with the corresponding peptide-G-KLH (concentration of the peptide was 100 or 400 µM) abolished specific labeling (Fig. 1D,G). Furthermore, the rabbit or goat preimmune serum used on adjacent sections at the same concentration as AmOA1 antiserum did not show any staining (Fig. 1B,F).

The AmOA1 peptide used for immunization in rabbit shows 87% sequence identity to the fruit fly OAMB receptor isoform A peptide sequence (NH_2_-DFRFAFKRIICRCFC-OH; amino acids 578–592 of DmOA1 A; AAF55798). Therefore, we used the *Drosophila oamb* ^96^ mutant line, which contains a deletion spanning exons 2 and 3 and does not produce the DmOA1 A protein in the brain [44], as a control for antibody specificity. We used Ranti-AmOA1 to label sections from brains of wild type Canton S (CS) and *oamb^96^* flies (Figure S1H–K). As expected [40], the Ranti-AmOA1 antibodies labeled cells and neuropils in the mushroom body alpha prime and beta prime lobes as well in the antennal lobes of CS female flies (Fig. S1H,J). However, specific labeling in the mushroom body lobe and antennal lobe was absent from the *oamb^96^* sterile female flies (Fig. S1I,K). These data show that the antibodies specifically recognized the *Drosophila* OAMB receptor isoform A.

### Neuroanatomical distribution of the AmOA1 immunostaining in the honey bee brain

Our description below of AmOA1 distribution in the brain is based on immunostaining of ten honey bee forager brains processed with Ranti-AmOA1 and five honey bee brains processed with Ganti-AmOA1. A schematic view of the overall AmOA1 immunoreactivity of cell bodies and processes in the honey bee brain is shown in Figure 2. This drawing was made from confocal images of frontal sections of bee brain. It summarizes the most typical labeled cell bodies and processes in the central complex, mushroom bodies, antennal lobes, lateral protocerebrum, optic lobes and subesophageal ganglion. We begin each section below with a brief description of the anatomy of that area of the brain.

### AmOA1 staining in the antennal lobes

**Figure 3:**
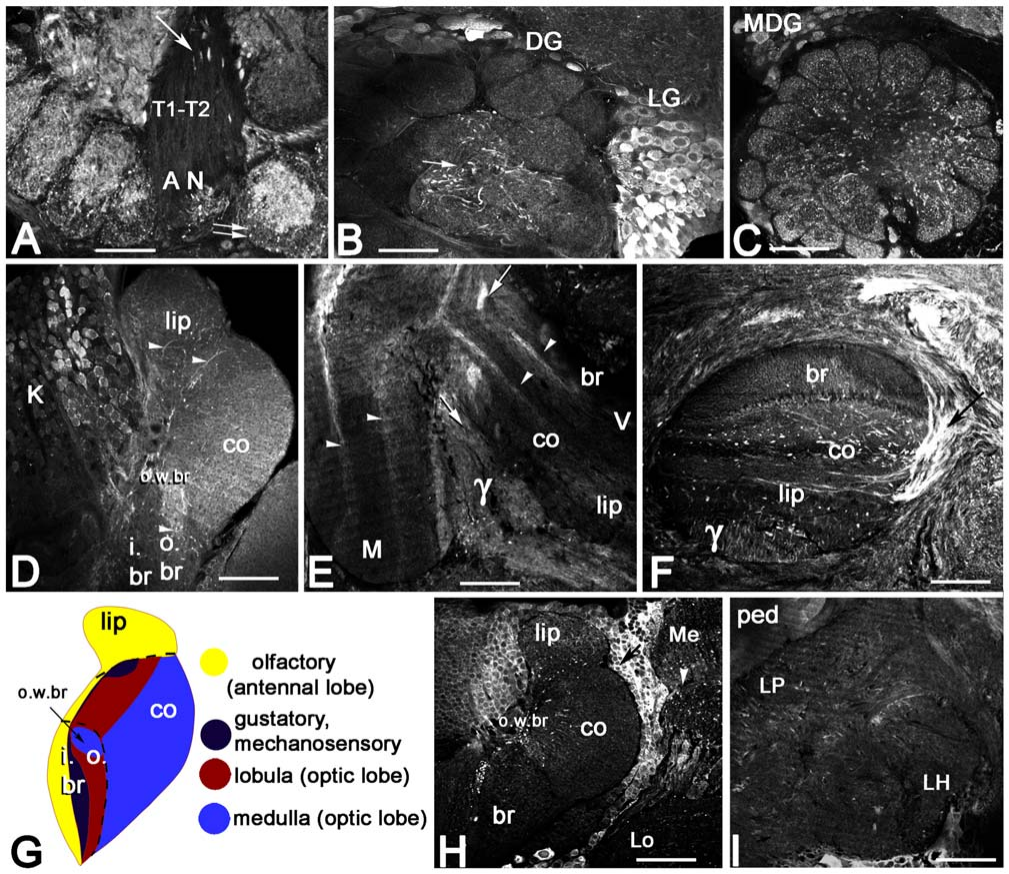
AmOA1 is expressed in the antennal lobes, mushroom body and lateral protocerebrum. **A–C**: Example of cell bodies and neuropil labeled with AmOA1 in the antennal lobe. The lateral side of the antennal lobe is on the right. **A**: In the antennal nerve (AN), the tracts of olfactory receptor neurons (T1–T2) have a few beaded processes positive for staining with AmOA1 antibodies (arrow). The glomeruli surrounding the entrance of the antennal nerve show different patterns of AmOA1 immunoreactivity. The double arrows point out an example of low intensity AmOA1 staining in the glomerular cortex where the endings of the olfactory receptor neurons are located. In contrast, the core region of this glomerulus has a high level of AmOA1 staining. **B**: Neurons in the lateral group (LG) exhibit a high intensity of AmOA1 immunoreactivity in their cell bodies and in their axons that are in the coarse area (arrow) of the antennal lobes. The cells bodies of the dorsal group (DG) also show intense staining. **C**: The medial dorsal group (MDG) of antennal neurons was also stained with high intensity with anti-AmOA1 antibodies. **D–H**: AmOA1 immunoreactivity in the mushroom body. **D**: One half of a calyx stained with AmOA1 antiserum. Kenyon cell bodies (K) that supply the collar or outer basal ring exhibit differing AmOA1 immunoreactivity. The dendrites of Kenyon cells are labeled with high intensity in the outer wedge basal ring zone of the calyx (o.w.br) whereas less intense staining is observed in the calyx collar (co). Arrowheads indicate endings of extrinsic (feedback) neurons expressing AmOA1 in the calyx lip, the outer basal ring (o.br), and with a few branches in the collar neuropil. The inner basil ring (i. br) show relatively low levels of staining. **E**: A sagittal section made through the mushroom body vertical (V) and medial (M) lobes. Dorsal is at the top. In the medial and vertical lobes, there are two layers of Kenyon cell axons stained with anti-AmOA1 antibodies (arrowheads). These layers correspond to the basal ring and collar Kenyon cells. The class II clawed Kenyon cells, which have their axons in the ventral part of the vertical lobe (the γ lobe), are also positive for AmOA1 expression. Arrows show the axons of γ lobe Kenyon cells brightly labeled with anti-AmOA1. The cell bodies of these cells lay outside of calyx (see arrow in **H**). **F**: A frontal section through the vertical lobe of mushroom body. AmOA1 immunoreactive extrinsic neurons enter and branch in the γ lobe. The arrow indicates an example of AmOA1 immunoreactive neurites of feedback neurons that enter in the vertical lobe. **G**: A schematic representation of a calycal cross section illustrating calycal regions receiving olfactory (yellow), gustatory and mechanosensory (dark blue), and visual input from the lobula (dark red) and medulla (light blue). **H**: The AmOA1 immunoreactive cell bodies of neurons located in a cluster between the dorso-medial edge of the medulla (Me) and the lateral calyx are neurons that connect the mushroom body calyx with the medulla. The cells in this cluster have fibers in the anterior superior optical tract (asot). The arrowhead shows axons from these neurons entering the medulla serpentine layer. AmOA1 positive fibers run along the dorsal edge of the lobula (Lo) and enter into the serpentine layer of the medulla. A few AmOA1 positive fibers enter through the lobula optical tract (lot) that connects lobula and mushroom bodies. **I**: The lateral protocerebrum (LP) exhibits few fibers or stained structures (midline is on the left). There are diffuse processes in the lateral horn (LH) that express AmOA1 in this animal. Scale bar: 50 µm in A,B,C; 25 µm in D,E,F,H; 75 µm in I.

The primary olfactory neuropil is the antennal lobe, which consists of approximately 160 spherical glomeruli that surround a coarse neuropil in the core (Fig. 2, 3A–C, 5). The principal inputs of the glomeruli are olfactory receptor neurons from the antennal nerve. Glomeruli are also invaded by processes from at least three cell types: (1) two types of projection neurons; uniglomerular projection neurons, which connect the antennal lobe with the mushroom body calyx and the lateral horn via the lateral or medial antennocerebral tract (the l- and m-ACT), and multiglomerular projection neurons that connect the antennal lobe with the protocerebral lobe and lateral horn via the medio-lateral tract (mlACT); (2) two or more types of local interneurons with axons in the coarse neuropil and dendrites in the glomeruli; and (3) multiglomerular interneurons that express biogenic amines and have cell bodies outside of the antennal lobe, for example VUMmx1 [34], [52]–[54]. All but the latter type of interneuron have cell bodies in one of three groups that lie lateral (LG), dorsal (DG) or mediodorsal (MDG) to the antennal lobe (Fig. 3). Each group (LG, DG, MDG) contains the cell bodies of two or more types of antennal lobe neurons.

AmOA1 immunoreactivity was present in all three cell body groups (MDG, DG, and LG) that surround the glomerular neuropil as well as in glomeruli and the coarse (central) area of the antennal lobe neuropils (Fig. 3A, B, C). The olfactory receptor neurons enter from the antennal nerve via four tracts (T1–T4), but only T1 and T2 enter the coarse area in the center of the antennal lobe (Fig. 3A). The olfactory receptor neuron axons were not stained with the AmOA1 antibodies, except for a few scattered beaded processes, which may also be glial cells, in the T1–T2 tracts where they enter the coarse area (Fig. 3A arrow). The glomeruli, as well as the coarse neuropil of antennal lobes, were strongly labeled with AmOA1 antibodies (Fig. 3A–C).

Among the different areas of the antennal lobe, labeling in the glomeruli was the most variable both within and between preparations. Immunostaining in some glomeruli revealed a very strongly labeled core region with absence of staining in the surrounding cortex (Fig. 3A double arrow) where most of the olfactory receptor neurons from sensory tracts T1–T3 send their input to the glomeruli [55]. Some glomeruli exhibited an almost complete absence of AmOA1 immunoreactivity, or staining was scattered throughout the entire glomerulus, (Fig. 3B). In other glomeruli the cortex area was stained with higher intensity (Fig. 3C).

One type of AmOA1 labeled process in the coarse neuropil corresponds to axons from a large group of cells located laterally (LG; Fig. 3B). Among the types of antennal neurons, the LG contains cell bodies of local interneurons [53]. A large fraction of neurons from cluster LG were strongly labeled by AmOA1 antiserum in the cell bodies and axons in the coarse neuropil area. Cell compartments stained with different intensity. The nucleus was not labeled. Bright staining observed in the cytoplasm could be due to labeling of receptors during translation, in the process of being transported to the plasma membrane, during receptor recycling and/or receptors targeted for degradation.

The DG and MDG (dorsal and medio-dorsal groups respectively, Fig. 3B, C) also contained AmOA1 immunopositive neurons. These neurons may correspond to multi-glomerular projection neurons given the presence of staining in the medial-lateral tract (not shown), which connects the antennal lobes with the protocerebral lobe [54]. AmOA1 staining is absent in the axons of the projection neurons of the m- and l-ACT. In our preparations staining in the target neuropil of these neurons in the lip and inner basal ring zones of the calyx of the mushroom bodies has a low intensity. We have not been able to conclusively assign this weak staining to PN axon endings or to Kenyon cell dendrites (Fig. 3D, H). Double staining experiments with labeled PNs need to be performed in order to confirm these observations.

### AmOA1 staining in the mushroom bodies

In honey bees, each mushroom body consists of paired calyces (lateral and median) connected to a stalk-like structure that forms a peduncle with two (medial and vertical) lobes (Fig. 2). The major components of the mushroom body are intrinsic neurons known as the Kenyon cells [56]. Their axons form the pedunculus and lobes and their dendrites make up the calyces. There are two broad classes of Kenyon cells. The Class I Kenyon cells have cell bodies contained within the calycal cups and their dendrites extend into the inner wall of the calyx. Their axons project through the pedunculus and give rise to two branches, one in each lobe. Class II Kenyon cells, also referred to as “clawed” Kenyon cells, have soma that lie outside the calyces. Their neurites extend through the outer wall of the calyx where they produce distinctive “clawed” dendrites. Class II Kenyon cells have axons which supply the ventral part, or γ division, of the vertical lobe and may also bifurcate to supply the vertical and medial lobes [57]–[60].

The calyces are divided into modality specific zones – lip, collar and basal ring - that receive input from different sensory modalities [54], [60]–[63]. The lip receives input from olfactory projection neurons, the collar receives input from visual, tactile and gustatory processing neuropils, and the basal ring receives input from olfactory, gustatory, tactile and visual neuropils (see schematic in Fig. 3G).

It is important to note here that Kenyon cell bodies were labeled with different intensity across the calyx (Figures 3D,H, 7B, D2), which is in accordance with in situ analysis [43]. The AmOA1 antiserum labels a subpopulation of class I Kenyon cell bodies with higher intensity. It is possible to follow their stained axons and dendrites in the outer basal ring zone (Fig. 3D,H); the area at the edge of the dorsal basal ring region is strongly labeled in all preparations. The Kenyon cell dendrites in the collar are less intensely stained than dendrites in the outer basal ring (Fig. 3D,H). The edge of the outer basal ring area receives visual input from axons from the medulla, whereas the collar region receives input from both the medulla and lobula of the compound eyes. The Kenyon cells with dendrites in the collar and basal ring express AmOA1 protein in their cell bodies, dendrites and in their axons that make up the layers in the vertical lobe and medial lobe (Fig. 3E, F).

The class II Kenyon cells also exhibited strong immunoreactivity in their cell bodies and their axons in the ventral part, or γ division, of the vertical lobe (Fig. 3E, H). Some of these AmOA1expressing class II cells have their cell bodies outside of the mushroom body calyces (arrow in Fig. 3H) and their dendrites spread throughout the calyx into all sensory input zones. Their axons are brightly labeled in the γ division of the vertical lobe (Fig. 3E).

Apart from the Kenyon cells, other types of neurons in the mushroom body lobes also express the AmOA1 receptors. These are extrinsic neurons with large AmOA1 antiserum positive axons in the γ division of the vertical lobe. The neurons that enter the vertical lobe just above the γ division (arrow in Fig. 3F, also 7F, 7H2) correspond to the feedback neurons that connect mushroom body calyces with mushroom body lobes.

**Figure 4:**
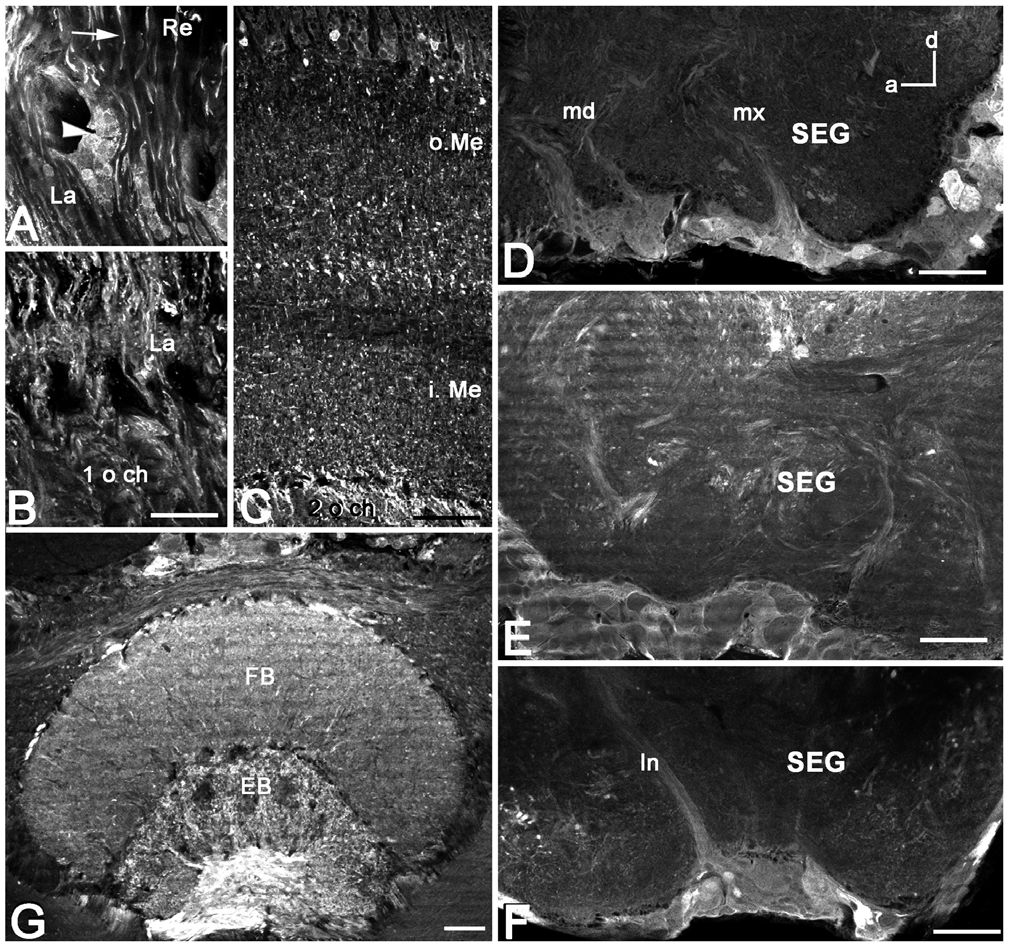
AmOA1 immunoreactivity is observed in the visual neuropil (A–C), central complex (D) and subesophageal (SEG) ganglion (E–G). **A:** In the lamina (La), AmOA1 immunoreactivity was observed in the ends of some photoreceptor cells that originate in the retina (Re; arrow) and in a subset of the monopolar cells (arrowhead). **B:** AmOA1 expression is seen in processes in the first chiasma (1 o ch) and the lamina neuropil. **C:** AmOA1 immunoreactive processes are organized in discrete strata in the medulla (Me). The columnar intrinsic neurons are intensely stained. The serpentine layer, which separates the outer medulla (o. Me) from the inner medulla (i. Me), has staining in tangential elements that may belong to medulla neurons that are connected to the calyx of the mushroom bodies. The second optic chiasma (2 o.ch) between the medulla and lobula also contains AmOA1 immunoreactive processes. **D:** A sagittal section made through the midline of the subesophageal ganglion where the VUM neurons are located in clusters in the maxillar (mx) and mandibular (md) neuromeres is shown. Anterior (a) is to the left and dorsal (d) is on the top. AmOA1 staining is observed in neurons with small cell bodies located in the clusters with the VUM cells; the VUM cells themselves are not stained with anti- AmOA1 antibodies. **E, F:** Frontal sections of the subesophageal ganglion from two different bee preparations. AmOA1 staining is observed in small cell bodies located in the midline of the ventral part of the subesophageal ganglion. ln, lateral neurite tract. **G:** The fan-shaped (FB) body is less intensely stained with anti-AmOA1 than the ellipsoid body. Scale bars: 50 µm in A; 25 µm in B,C,G; 70 µm in D,E,F.

The areas of the calyx that receive inputs from the antennal lobe (lip and inner basal ring) stained with somewhat different levels of intensity in different animals (Fig. 1A, 3D, 3H, 7B, 7D2). For example, the lip is more intensely labeled in figure 3H than in 3D. In most preparations, the lip and inner basal ring were less intensely stained compared to subdivisions of the calyx that receive input from visual processing areas (collar and outer basal ring; Fig. 3D, 3G, 7B, 7D2). Especially brightly stained was the edge area in the outer zone of the basal ring (o.w.br in Fig. 3D, 7B). This area corresponds to rosette-shaped Kenyon cells that receive input from the ventral medulla [60], [61]. The stained processes in this outer basal ring area may correspond to the endings of afferents from the medulla (Fig. 3G,H). Neurons that connect the medulla to the mushroom body calyx via the anterior superior optical tract (asot) are located in the posterior edge between the mushroom body calyx and the visual neuropil (Fig. 2, arrowhead in 3H). Some of the neurons from this group showed strong AmOA1 immunoreactivity in cell bodies and axons in the asot (Fig. 2), processes in the serpentine layer of the medulla (Fig. 4C), and in the visual input area in the outer basal ring (Fig. 3D,H). There is also staining in some neurons that connect the mushroom bodies with the lobula through the lobula optic tract (lot; Fig. 2).

In most preparations, the protocerebral lobe is stained with AmOA1 antibodies at a lower intensity compared the antennal lobe and mushroom body neuropil (Fig. 3I). The few positively stained processes are scattered through the lateral protocerebrum neuropil and in the lateral horn.

### AmOA1 expression in the optic lobes

The optic lobes are neuropilar structures adjacent to the compound eyes. They receive and process input from photoreceptors originating in the retina (Fig. 2). The optic lobe consists of the lamina, the medulla (the outer and the inner separated by the serpentine layer; Fig. 2, 3H, 4A–C), and the lobula (Fig. 3H). Some photoreceptor cells exhibit intense staining with the AmOA1 receptor antibodies (Fig. 2, cluster 3; arrow in Fig. 4A). Lamina monopolar cells express the AmOA1 receptor as illustrated in figure 4A where a cluster of monopolar cell bodies is brightly stained with AmOA1 antibodies (Fig. 2, cluster 4; Fig. 4A arrowhead). The first and second optic chiasmata between the lamina and the medulla, and between the medulla and the lobula, respectively, have AmOA1 immunoreactive fibers (Fig. 4B, C). In the medulla there are columnar cells labeled with AmOA1 antiserum (Fig. 2, cluster 5; Fig. 4C) that may either be intrinsic to the medulla or that may be transmedullary and connect the medulla with the lobula via the 2nd optic chiasma. The AmOA1 antiserum stained processes in the medulla serpentine layer may belong to the cells that connect the medulla to the mushroom body calyces (Fig. 2, cluster 2; Fig. 3H), the cell bodies of which are located between the base of the lateral calyx and the dorso-medial edge of the medulla. Likewise, stained processes in the lobula may belong to cells that connect the optic lobula with mushroom body lobula cells (Fig. 2).

### AmOA1 staining in the subesophageal ganglion

The subesophageal ganglion of the honey bee is composed of the fused mandibular, maxillary and labial neuromeres. These neuromeres receive gustatory sensory information and are involved in the motor control of the mouthparts [64]. There are clusters of small cell bodies located in the median ventral and lateral parts of the subesophageal ganglion that stained strongly with AmOA1 antibodies (Fig. 2, cluster 7; Fig. 4D–F). The VUM cells, characterized by large cell bodies in the median part of the subesophageal ganglion, are not stained in either the cell bodies or in the primary neurites in the median and lateral tracts (Fig. 4D–F). Scattered processes staining with AmOA1 antiserum are present in the subesophageal ganglion neuropil and may belong to the descending and ascending neurons as well as to local interneurons (Fig. 4D–F). Staining was absent in the mandibular labial and maxillary nerves (data not shown).

### AmOA1 staining in the central complex

The central complex is a prominent structure located in the central brain between the two protocerebral hemispheres [65], [66]. It comprises four neuropilar regions: the ellipsoid body (the lower division of the central body), the fan shaped body (the upper division of the central body), the paired noduli and the protocerebral bridge. These structures are interconnected by sets of columnar neurons that form a regular projection pattern.

Anti-AmOA1 staining in the honey bee central complex is illustrated in Figures 2 and 4G. A group of AmOA1 expressing cells located above the dorsal lobe in the medial protocerebrum (Fig. 2, cluster 8) send their axons to terminate in the ellipsoid body (Fig. 4G), and these axons have a very high level of AmOA1 immunoreactivity. These axons can be seen entering the base of the ellipsoid body in Fig. 4G, and we traced them back to the cell bodies located near the dorsal lobe (Fig. 2, cluster 8; confocal image not shown). The fan shaped body neuropil has a lower level of AmOA1 immunoreactivity relative to the high intensity staining in the ellipsoid body (Fig. 4G).

### GABAergic local interneurons in the antennal lobes label with AmOA1 antibodies

**Figure 5:**
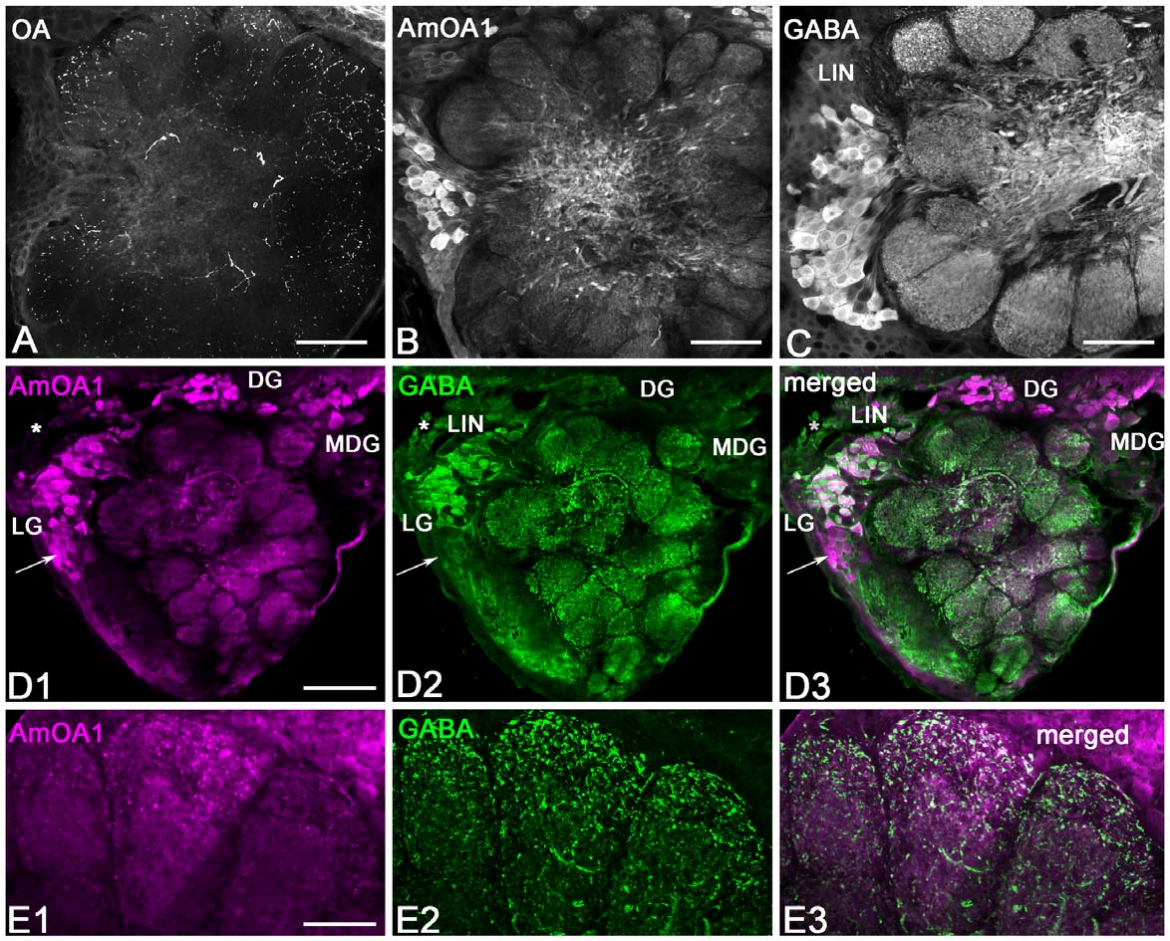
A subset of the GABAergic local interneurons expresses the AmOA1 receptor in the antennal lobe. **A**: In a frontal section of the antennal lobe, octopamine (OA) immunoreactive processes originating from VUM (ventral unpaired median) neurons invade each glomerulus of the antennal lobe, the middle is on the left. **B**: In the adjacent antennal lobe section, anti-AmOA1 antibodies revealed staining in the cell body cluster lying laterally and providing AmOA1 positive fibers to the non-glomerular (coarse) area of the antennal lobe. **C**: GABA antiserum used on a frontal section of the antennal lobe revealed GABA immunolabeled local interneurons (LIN) that have their cell bodies located laterally. These cells project axons into the coarse area of the antennal lobe and have fine arborizations ending in each glomerulus. **D**: Double staining with anti-AmOA1 and anti-GABA revealed a subset of the lateral group (LG) of AmOA immunoreactive cells and processes (magenta, **D1**) co-localize with GABA (green, **D2**). In the merged image (**D3**) co-localization appears white. Not all lateral group neurons (arrow), or the dorsal and medial-dorsal group neurons (DG and MDG) positive for AmOA1 express GABA. Conversely, not all GABAergic cells express AmOA1 (asterisk). **E1-3**: Enlarged images of the antennal glomeruli double stained with anti-GABA and anti-AmOA1 antibodies. The enlarged images of the antennal lobe glomeruli reveal that the AmOA1 immunoreactivity is in beaded profiles (**E1**). The GABA-like neurons (**E2**) have much more elaborate and widespread branching than AmOA1 staining (**E3**). Scale bars: 25 µm in A, B, C, 50 µm in D, 15 µm in E.

In order to compare the distribution of octopamine and the distribution of AmOA1 in the antennal lobes, we labeled adjacent sections of the same brain with octopamine (Fig. 5A) and AmOA1 antisera (Fig. 5B). The octopamine-like immunoreactive processes from VUM neurons enter in the coarse center of the antennal lobes from the ventro-posterior deutocerebral area and give rise to very fine branches to supply each glomerulus of the antennal lobe. Staining of the adjacent section with AmOA1 antiserum reveals cell bodies clustered in the lateral rind of the antennal lobe with AmOA1 positive profiles in the central coarse area and fine distribution of AmOA1 positive varicosities in each glomerulus (Fig. 5B). These data suggest that octopamine is released parasynaptically, rather than presynaptically to each profile. The cell bodies in the lateral cluster of the antennal lobe labeled with AmOA1 antibodies (Fig. 5B,D1) belong to local interneurons that are known to be GABA positive [67] (Fig. 5C,D2). The anti-GABA antibody stained local interneurons in the cell bodies, processes in the coarse area of the antennal lobe, and highly packed processes in each glomerulus (Fig. 5C, D2). Comparison between the distribution of AmOA1 (Fig. 5B, D1) and GABA (Fig. 5C,D2) revealed that GABAergic local interneurons express the AmOA1 receptor. All co-localized cell bodies and processes are white in the merged image (Fig. 5D3). At higher magnification, the GABA-like immunoreactive neurons have much more elaborate and wide spread branching than AmOA1 staining (Fig. 5E), which could reflect very localized expression of the AmOA1receptor on GABAeric cell processes.

In our preparations, the majority of the GABAergic neurons express AmOA1 receptors with a range of staining intensity. However, there are some GABAergic neurons where the staining for AmOA1 is very low or absent (e.g. Fig. 5D, where a group of cells labeled with an asterisk have very low AmOA1 immunoreactivity). Comparisons of GABAergic with AmOA1 staining in the cell bodies and processes reveal that not all GABA neurons express the receptors in those areas of the cell (Fig. 5E1-3, Figure S1D–F).

Conversely, some AmOA1 positive neurons are not GABAergic. The arrow in figure 5D indicates AmOA1 receptor positive neurons in the lateral cluster that are not stained with the GABA antibody. In addition, the AmOA1 receptor is expressed in cell bodies of the medial cluster (DG and MDG) that do not show GABA-like immunoreactivity.

### A subpopulation of mushroom body feedback neurons express AmOA1

**Figure 6:**
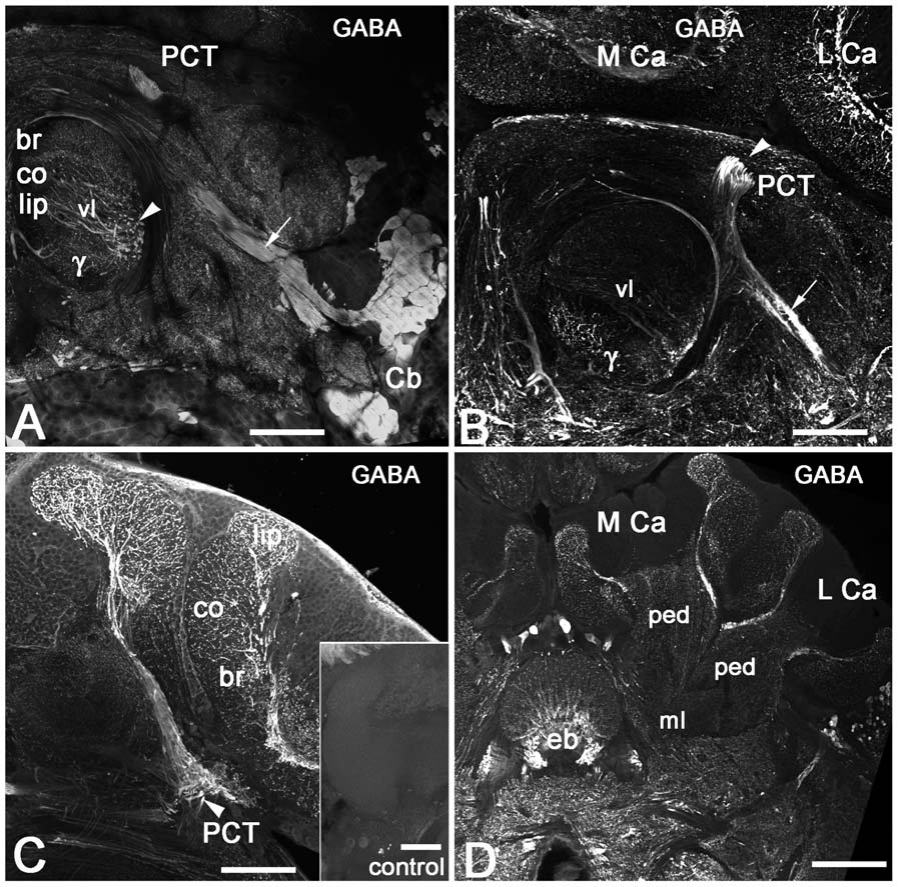
GABAergic feedback neurons in the protocerebro-calycal tract (PCT) are shown. **A**: GABA-like immunoreactive neurons with large cell bodies (Cb) are located anteriorly and laterally between the lateral protocerebrum and optic lobes. Each cell sends its primary neurite through the lateral protocerebrum towards the vertical lobe of the mushroom body forming the large GABA-like immunoreactive fiber tract (arrow in **A**, **B**). **B**: After reaching the vertical lobe of the mushroom body, each primary neurite divides into two branches. One branch enters the vertical lobe producing fine arborizations in the dorso-lateral margin of the vertical lobe (vl, arrowhead in **A**). The other branch follows the GABA-like immunoreactive fibers (arrowheads in **B**, **C**) through the protocerebro-calycal tract (PCT) to enter the calyces (**C**, **D**). **C**: Each calyx receives GABA-like immunoreactive fibers that produce fine arborizations in the lip, collar (co) and basal ring (br). **D**: GABAergic feedback neurons innervate the median (M Ca) and the lateral (L Ca) calyces, pedunculus (ped) and medial lobe (ml). The insert in **C** illustrates the specificity of the GABA antibodies. This section shows the lack of staining when the GABA antiserum was preadsorbed with 0.01 mM GABA conjugated to carrier protein (BSA) prior to use on the section. Scale bar: 100 µm in A, B; 50 µm in C, D.

A group of GABAergic neurons provide feedback from the mushroom body output lobes and pedunculus to the calyces [67], [68] (Fig. 6A–D). These GABA stained feedback neurons have their ventral somata clustered in the anterior lateral protocerebral lobe (Fig. 6A). Their primary neurites project dorsally and medially and then bifurcate at the dorso-lateral margin of the vertical lobe. One branch enters the vertical lobe and innervates the medial lobe and pedunculus (Fig. 6A–D; only the beginning of medial lobe is shown in Fig. 6D). The other branch runs outside the mushroom body within the protocerebral-calycal tract (PCT) in a dorsal and posterior direction, bifurcates between the median and lateral calyces and then sends collaterals into inner ring tracts that run between the peduncular stalk and calyx (Fig. 6C,D). Each feedback neuron innervates both the median and the lateral calyx [69].

**Figure 7:**
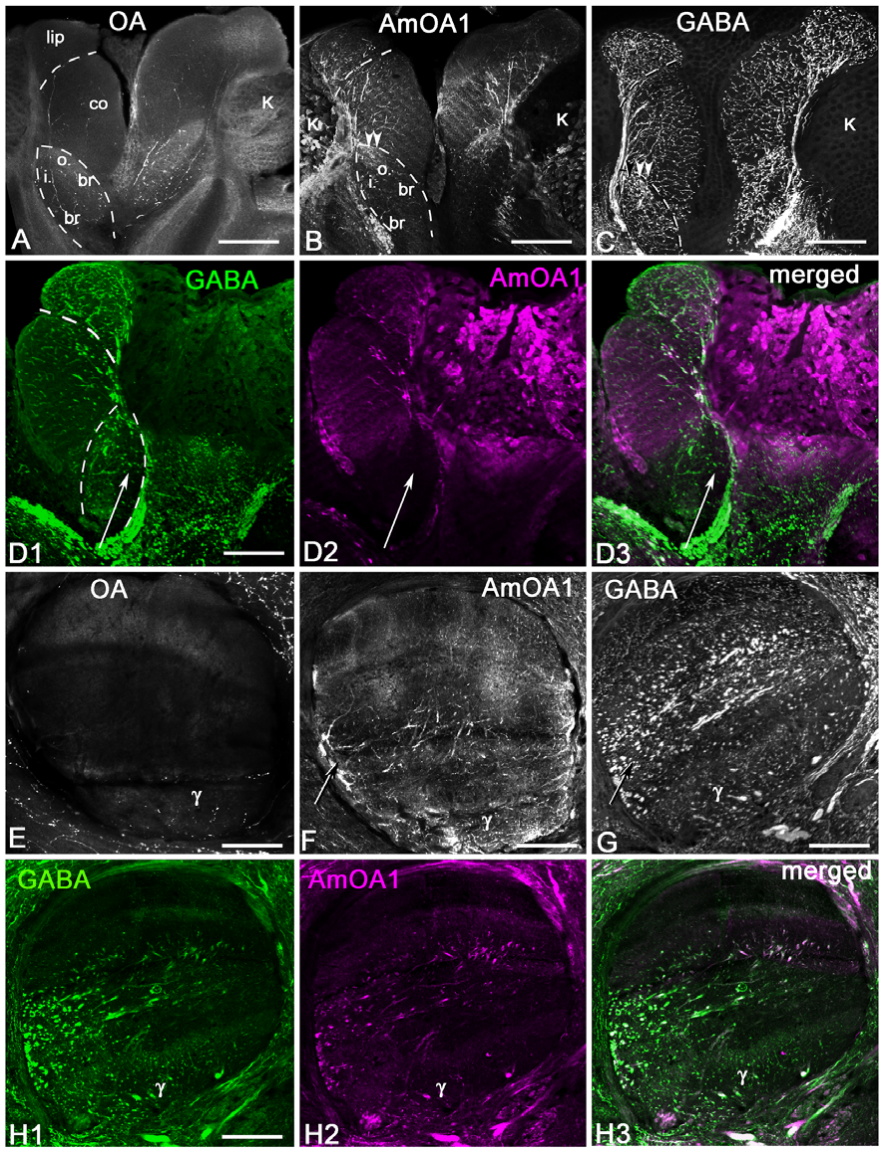
A subset of the GABAergic feedback (PCT) neurons in the mushroom body expresses the AmOA1 receptor. **A**: Frontal section of the halves of two calyces labeled with octopamine antiserum (OA). Octopamine immunoreactive processes are in the outer and inner basal ring (o. br, i. br), collar (co) and calyx lip. **B**: The frontal section adjacent to the section shown in **A** labeled with anti-AmOA1 antibodies. The AmOA1 positive processes are in the lip, collar and basal ring zones of the calyx in large afferent profiles. Note that the Kenyon cell bodies (K) of the outer basal ring and collar are also labeled with anti-AmOA1. The double arrowheads indicate the outer wedge of the basil ring, which shows relatively intense staining. **C**: GABA-like immunoreactivity in the feedback neurons have ends in all zones of the calyx. **D1-3**: Double staining for GABA and AmOA1 in frontal sections of the lateral calyx. Not all of the GABAergic feedback neurons exhibit anti-AmOA1 immunolabeling (arrow). The AmOA1 receptor mostly co-localized with feedback neurons that end in the lip and basal ring zone of the calyx. **E**: Frontal section through the vertical lobe of the mushroom body labeled with octopamine antiserum. The vertical lobe has octopamine immunoreactive processes only in the γ lobe and in the neuropil of the protocerebrum that surrounds the vertical lobe. **F**: In a section adjacent to the section shown in **E**, the vertical lobe of the mushroom body labeled with AmOA1 antiserum. The anti-AmOA1 staining is in: i) extrinsic neurons branching in the γ lobe and ii) processes from feedback neurons that enter the lobe laterally (arrow). The axons of Kenyon cells in the γ lobe labeled with low intensity. **G**: Frontal section of the vertical lobe labeled with GABA antiserum. The feedback neurons exhibit GABA-like immunoreactivity in profiles that enter in the lobe medio-laterally and branch in the lip, collar and basal ring zone of the vertical lobe. The extrinsic GABA-like immunoreactive processes branch in the γ lobe. **H1-3**: Double staining with anti-GABA and anti-AmOA1 in the section adjacent to the section shown in **G**. A subset of GABAergic feedback profiles co-localized with anti-AmOA1. Scale bar: 25 µm.

In order to compare the distribution of the AmOA1 receptor in the mushroom body with the distribution of octopamine, we stained adjacent sections with AmOA1 or octopamine antisera (Fig. 7A,B,E,F). Consecutive sections in Figures 7A and B reveal octopamine and AmOA1 receptor in the lip and outer basal ring zones. The octopamine immunoreactive profiles belong to the VUMmx1 and VUMmd1 neurons, which have their cell bodies in the subesophageal ganglion. The vertical lobe has octopamine immunoreactive processes only in the γ lobe and in the neuropil of the protocerebrum that surrounds the vertical lobe (Fig. 7E). Interestingly, AmOA1 expression is more widespread than octopamine in the vertical lobe (Fig. 7F).

AmOA1 receptor immunoreactivity is in the arborizations of processes that arrive in the calyx through the PCT tract, which contains GABAergic feedback neurons (Fig. 2, cluster 1, Fig. 6). These GABAergic feedback neurons branch in all areas of the calyx, which includes the lip, collar and basal ring, as well as into the vertical and medial lobes (Fig. 7C, D1, G, H1). In our immunofluorescence double labeling with anti-GABA and anti-AmOA1 antibodies, subsets of GABAergic PCT neurons co-localized with the AmOA1 receptor (Fig. 7D,H).

The feedback neurons that express AmOA1 immunoreactivity in the calyx are in the lip, collar and outer basal ring area (Fig. 7B, D2). There is low intensity staining in the inner basal ring area, with high intensity of staining at the edge of basal ring zone (double arrowheads in Fig. 7B). The arrows in figure 7F,G show where feedback neurons enter the vertical lobe (the midline of the brain is on the left). Double staining on the same sections with anti-GABA and anti-AmOA1 revealed only a small subpopulation of GABAergic feedback neurons in the calyx and vertical lobes co-stained (Fig. 7D3,H3). These subpopulations of GABA and AmOA1 immunoreactive feedback neurons have their endings mostly in the lip, collar and outer layer of the basal ring (Fig. 7D3) while the inner basal ring zone is not stained with AmOA1 antibodies. In the vertical lobe, the axons of feedback neurons that express AmOA1 receptors are mostly in the region occupied by the axons of Kenyon cells with dendrites in the collar and lip zones of the calyx. In addition, there are extrinsic GABAergic neurons that enter the γ lobe that also co-stain with anti-AmOA1 (Fig. 7F, H2, H3).

### The tangential GABAergic neurons of the central complex express AmOA1

**Figure 8:**
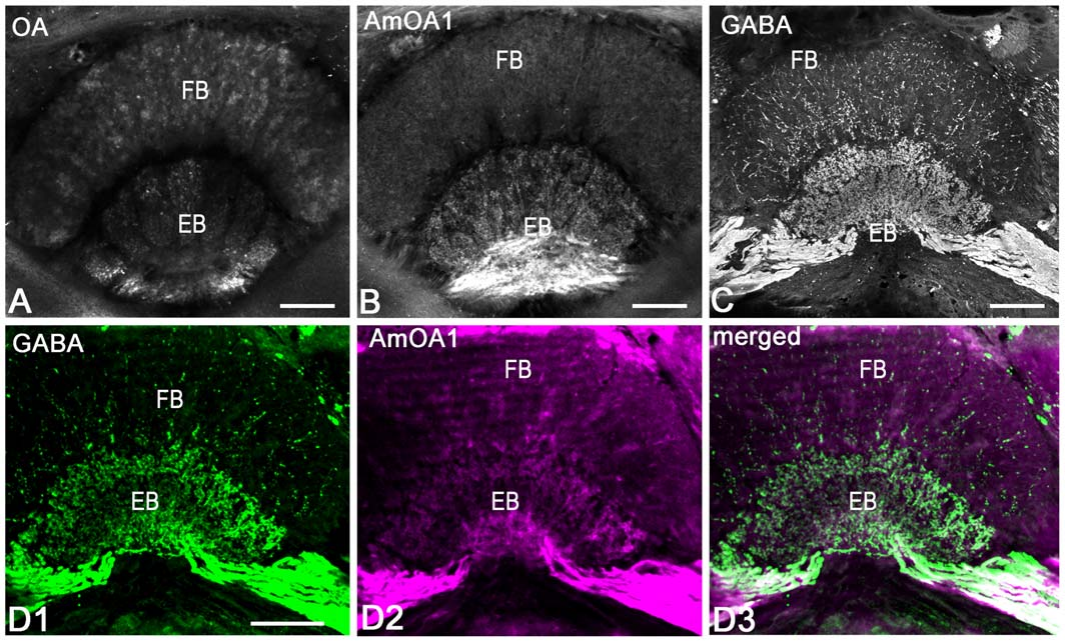
Tangential GABAergic neurons in the central complex express the AmOA1 receptor. **A**: A frontal section of the central complex labeled with anti-octopamine (OA). Octopamine in is present in the ellipsoid body and the fan shaped body. **B**: Frontal section adjacent to the section shown in **A** labeled with anti-AmOA1 antibodies. The AmOA1 immunoreactivity is in the tangential fibers that invade the ellipsoid body. Note the fan shaped body is less intensely stained than the ellipsoid body. **C**: GABA-like immunoreactivity in the tangential neurons innervating the ellipsoid body and fan shaped body. **D**. Section adjacent to section **C** double stained with GABA antiserum (**D1**, green) and anti-AmOA1 antibodies (**D2**, magenta) revealed that GABAergic processes in the ellipsoid body are co-labeled with AmOA1 receptor (white in merged images) in **D3**. Scale bar: 25 µm.

We used octopamine antiserum to compare the distribution of octopamine labeling with AmOA1 receptor staining in the central complex of the honey bee on two adjacent sections (Fig. 8A, B). The origin of the octopamine innervations in the fan shaped body and ellipsoid body has several sources: neurons from clusters G4 and G2, and possibly some fibers from the VUM neuron(s) located in the subesophageal ganglion [32]. The section adjacent to the section shown in figure 8A was labeled with AmOA1 antibodies (Fig. 8B), and shows high intensity staining in the ellipsoid body with less intense staining in the fan shaped body. The neurons that stained for AmOA1 belong to the tangential GABAergic neurons whose cells bodies are located in cluster 8 (Fig. 2) and that send their input into the ellipsoid body and fine branches into the fan shaped body (Fig. 8C). The expression of AmOA1 in these GABAergic neurons was confirmed using double immunofluorescence staining with anti-GABA and anti-AmOA1 (Fig. 8D1, 2). The overlap of staining for GABA and AmOA1 confirms that AmOA1 receptors are expressed in the GABAerigic tangential neurons (Fig. 8D3).

### Expression of AmOA1 in other areas of the brain

The AmOA1 antiserum identified cells in other areas of the brain that we have not analyzed in detail and which need to be the focus of future study. It stains a group of median neurosecretory cells (MNC) in the pars intercerebralis (Fig. 2, cluster 9). These neurons project through the corpora cardiaca nerve II in the corpora cardiaca. Finally, photoreceptors in the ocelli also express the AmOA1 receptor (Fig. 2, cluster 6).

## Discussion

We used two antibodies against AmOA1 receptor raised in rabbit and goat against two distinct peptides to characterize the pattern of expression of a biogenic amine receptor (AmOA1) protein in the honey bee brain. In general, immunocytochemistry with anti-AmOA1 antibodies revealed labeling of cell body clusters throughout the brain together with their profiles in the neuropil (Fig. 2). Staining in dendritic and axonal profiles is consistent with expression of the receptor on membranes in those areas. Staining in or close to cell bodies could be due to expression of functional receptors on the cell body membranes, which would be consistent with parasynaptic release of octopamine [70]. It could also be due to trafficking of receptors for transport either to the periphery for insertion into the membrane or from the periphery for degradation. The expression pattern is consistent with results from in situ hybridization [43], which showed the presence of *Amoa1* transcripts in the Kenyon cells, cells in the optic lobes and the deutocerebrum with highly variable levels of expression. The widespread expression of the AmOA1 receptor is consistent with a role for this receptor in a number of behaviors modulated by octopamine.

### AmOA1 in the antennal lobe

**Figure 9:**
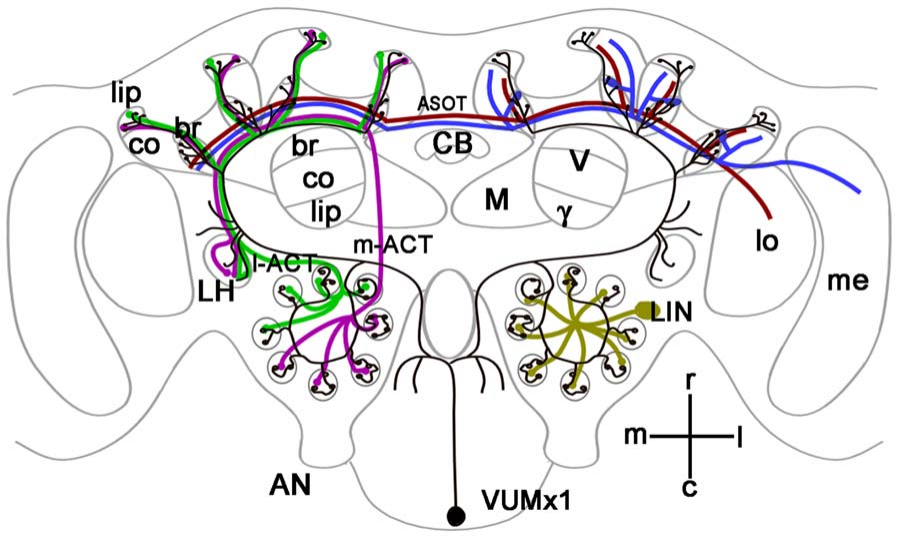
Schematic view of the visual and olfactory pathways in the honey bee mushroom body calyx. The figure was made after [34], [54], [60]–[63]. co, collar; br, basal ring; LH, lateral horn, l-ACT, lateral antenna-cerebral tract; m-ACT median antenna-cerebral tract; ASOT, anterior superior optic tract; AN, antennal nerve; CB, central body; M, medial lobe; V, vertical lobe; γ, gamma lobe; LIN, local interneurons; VUMmx1, ventral unpaired median neuron in maxillary neuromere; lo-lobula; me-medulla; m, median; l, lateral; r-rostral; c-caudal. Scale bar: 250 µm.

Behavioral studies demonstrating that RNAi mediated down regulation of AmOA1 expression leads to a reduction in olfactory associative learning in the honey bee [18] suggest that this receptor is an important part of the downstream targets of the associative reinforcement pathway mediated by VUMmx1. One way that AmOA1 may play a role in associative learning is through alteration of interactions in the antennal lobe network when odor is paired with sucrose [71]. In the antennal lobes, antibodies against AmOA1 stain three groups of neurons that are located laterally and dorsally. Double immunostaining with anti-AmOA1 and anti-GABA showed that these AmOA1 positive cells are a subpopulation of the GABAergic neurons. The neurons that stained with anti-AmOA1 but not with GABA might belong to subset of histaminergic local interneurons [72]. Local interneurons in the antennal lobes (LIN; Fig. 9) act to transform olfactory sensory information received from the antennae before it is sent via projection neurons to other areas of the brain. Expression of AmOA1 in the local interneurons provides a mechanism for linking the odor representation with the occurrence of the sucrose reward via release of octopamine from VUMmx1.

It is difficult to identify the subtype of neurons from the other two cell clusters in the antennal lobe (the dorsal and dorso-medial cell clusters) that stain with AmOA1. However, they are probably not the uniglomerular projection neurons because staining is absent from their axons and terminals in the lip and basal ring region of the mushroom body calyx, which are the targets of these projection neurons. (This observation will need to be confirmed by AmOA1 staining of uniglomerular PNs that have been intracellularly filled, as the receptor might be expressed in the dendrites of these neurons). On the other hand, AmOA1 immunoreactivity was present in the medio-lateral ACT (m-l ACT) and in the posterior lateral protocerebrum. This pattern suggests that some of the labeled neurons from the dorsal and dorso-medial groups may be multiglomerular projection neurons that receive input from several glomeruli [54].

Therefore, AmOA1 may not only affect odor representation via modulation of the local interneurons, but may also act upon the multiglomerular projection neurons that carry odor information from the antennal lobes to the lateral horn. This partitioning of AmOA1 mediated sensitivity to octopamine fits with a recent hypothesis that proposes different signaling roles for the two different olfactory tracts in the honey bee antennal lobes [54], [73]. In the fruit fly, a set of projection neurons that carry olfactory information directly from the antennal lobes to the lateral protocerebrum appear to be important for more stereotypical “experience-independent” behaviors [74]. Given the expression of AmOA1 in the m-l ACT, it would be interesting to determine if this receptor is involved in experience-independent olfactory behavior as well as playing a role in associative learning.

### AmOA1 expression in the mushroom bodies

Immunocytochemical studies suggest that octopamine is likely released in the mushroom body calyces and the γ division of the vertical lobe [30], [32]. The calyces are innervated by the VUMmx1 and VUMmd1 neurons, which have fine arborizations mostly in the lip and basal ring area, and a few branches are also present in the collar region [30], [32], [34], [35]. AmOA1 immunoreactivity overlapped substantially with VUM-based octopaminergic inputs in the mushroom body calyces.

The clawed Kenyon cells express AmOA1 in their cell bodies, dendrites and axons. These cells are of interest for several reasons. First, the axons of clawed Kenyon cells from across the entire calyx converge on the γ lobe of the mushroom body, thereby integrating input from different sensory modalities [60]. Second, different octopaminergic modulatory pathways, which may carry different types of information, target different regions of clawed Kenyon cells. Octopamine released from VUM neurons in the lip and inner basal ring likely targets dendrites of the different types of Kenyon cells. Octopamine released from VUM neurons probably also targets afferent neurons from visual neuropils and GABAergic neurons in the PCT since these neurons express AmOA1 receptors. In addition, the octopamineric VCBN neuron (nomenclature of [75]; LV from [32]) has a cell body located in the VUM cluster in the subesophageal ganglion, and its axons target axons of the clawed Kenyon cells in the γ lobe of the mushroom body. The fact that different types of sensory information from a number of different brain regions converge on clawed Kenyon cells, and that octopamine has been found to be necessary for appetitive olfactory learning [18]–[21], suggests that the clawed Kenyon cells should be an important subject for future studies.

A comparison of GABA immunoreactivity with the localization of staining for AmOA1 suggests that AmOA1 receptors are expressed in a subset of GABAergic feedback neurons (PCT). In the honey bee, most of the GABA immunoreactivity in the calyces arises from a group of feedback or recurrent neurons innervating both input and output areas of the mushroom bodies [68], [69]. These neurons change their properties in response to odors after olfactory conditioning and are likely to be involved in memory formation [69]. Our data provide the first evidence that the octopamine released in the mushroom body calyx and lobe may target GABAergic feedback neurons.

### Possible roles for AmOA1 in visual processing

In most preparations, the regions of the mushroom body associated with the visual calyx neuropil (collar and outer basal ring) showed relatively intense staining for AmOA1. We found small populations of class I Kenyon cells [60] that strongly express AmOA1 immunoreactivity in their cell bodies and in axons in both the vertical and medial lobes. These Kenyon cells have their dendrites in the visual calyx neuropil while their axons project through defined tracts in both the medial and vertical lobes to give rise to outputs from the mushroom bodies that represent the basal ring and collar regions. The edge area in the outer zone of the basal ring was especially brightly stained with AmOA1 antibodies. This area corresponds to rosette-shaped Kenyon cells that receive input from the ventral medulla [60], [61].

Octopamine has been shown to play various roles in visual processing in insects. In the honey bee, application of octopamine to the lobula enhances the directional antennal response to a moving striped pattern [8]. Furthermore, field potentials in the lobula showed increases in amplitude in the presence of exogenous octopamine [36]. In locusts, the release of octopamine by the PM4 cells (homologs of the honey bee G3A cells) is stimulated by multimodal input from the central brain [6]. This release of octopamine has been implicated in dishabituation of output neurons from the optic lobe [6], [76], [77]. Our results show that octopamine has multiple targets in the optic lobes, which suggests that the dishabituation may result from broad-based effects on the neural networks in the optic lobes.

### AmOA1 in the subesophageal ganglion

The subesophageal ganglion of the honey bee receives sensory projections from receptors located on the respective mouthparts through the corresponding mandibular, maxillary, and labial nerves [63], [64], [78]. It also gives rise to motoneurons that supply the muscles of the mouthparts. AmOA1 is expressed in the cells with small somata located in the lateral and median part of the ganglion. There are also AmOA1 positive processes in the neuropil of the subesophageal ganglion. However, it is difficult to assign a specific function to these AmOA1 positive cells. The lack of AmOA1 staining in the octopaminergic VUM cells suggests that this receptor is not acting as an autoreceptor to regulate the release of octopamine.

### AmOA1 in the central complex

The central complex is widespread in arthropods, and the complexity of its structure is correlated with the complexity of the organism's behavioral repertoire [66]. Mutation analysis in fruit flies has shown that the central body functions to control locomotor activity by affecting gait and the coordination of left and right sides of the body during walking as well as landmark orientation [79]. In various taxa, including the honey bee, the central complex receives inputs from the optic lobes and ocelli. Information from those modalities are represented, and sometimes segregated, in different subdivisions [65]. In the honey bee, electrophysiological recordings from neurons intrinsic to the central complex revealed that they respond to several stimulus modalities [80].

In the honey bee, octopamine immunoreactivity is found in the protocerebral bridge, in the chiasmal axons reaching forward through the fan shaped body, in the fan shaped body and in the ellipsoid body [32]. The AmOA1 receptor is expressed at relatively high levels in the ellipsoid body in the GABAergic tangential neurons that originate in the frontal part of the brain near the dorsal lobe. The protocerebral bridge stains much less intensely for AmOA1 with only a few labeled fibers running perpendicular to its columnar elements. Given that octopamine plays an important role in arousal [5], that octopamine affects the behavioral profile of the honey bee [16], and that the central complex is involved in control of motor behavior, it is possible that the AmOA1 receptor may be important for modulating arousal levels and coordinating motor behavior in the honey bee.

### Comparison of AmOA1 receptor and octopamine staining in the brain

Several studies have examined the pattern of octopamine-like immunoreactivity in the honey bee brain [30], [32]. Although, in general, the staining patterns for octopamine and the AmOA1 receptor overlap, there are regions where this is not the case. Regions where octopamine is present at relatively high levels while anti-AmOA1 antibodies show relatively low levels of staining, such as the lip of the mushroom body calyx [32], may be explained by the presence of other distinct octopamine receptors. Several other putative octopamine receptors exist in the honey bee [38]; however, to date, nothing is known about their expression pattern in the brain. More puzzling, perhaps, is the expression of AmOA1 in regions of the brain that appear to have little or no octopamine. For example, AmOA1 is present in processes in regions of the mushroom body vertical lobe, which do not show octopamine immunoreactivity [32]. These receptors may be responding to octopamine diffusing onto these cells from a relatively distant release site and, thus, acting as a neurohormone. Extrasynaptic signaling by a biogenic amine has been well characterized in *C. elegans* where dopamine acts upon motor neurons that express dopamine receptors, but which are not postsynaptic to dopaminergic neurons [81].

### Conclusions

AmOA1 receptors are expressed on GABAergic neurons located in several regions throughout the brain: the mushroom bodies, the antennal lobes and the central complex. Although it is clear that not all GABAergic neurons express the AmOA1 receptor, these data suggest that octopamine may modulate the activity of some inhibitory circuits via the AmOA1 receptor.

Although other octopamine receptors are probably present in the honey bee, they belong to a separate subfamily that most likely is linked to cAMP signaling [38], [42]. Thus, the expression pattern of AmOA1 presented here indicates cells that will respond to octopamine by increasing intracellular levels of Ca^2+^ [43]. This information will be a valuable guide in future studies examining the role of octopamine in specific behaviors.

## Supporting Information

Figure S1 Characterization of anti-AmOA1 antibodies and double staining controls. A: Double immunostaining of the bee antennal lobe with anti-GABA and Ranti-AmOA1 antibodies B, C: Two consecutive sections of the honey bee antennal lobe for control of sequential staining with Ranti-AmOA1 (B) and GABA (C) antisera where the GABA or AmOA1 antibodies were omitted. D-F: Double immunofluorescence staining in the bee antennal lobe with anti-GABA and anti-AmOA1 antibodies from goat (Ganti-AmOA1). The staining reveals that most GABAergic neurons in the lateral group (LG) are positive for AmOA1. Neurons that have low intensity staining with AmOA1 but a high level of staining with anti-GABA are shown by an asterisk. G1-G3: Comparisons of immunostaining of the anti-AmOA1 from rabbit (Ranti-AmOA1) with anti-AmOA1 from goat (Ganti-AmOA1) on the same section of the antennal lobe. Double fluorescence staining in the antennal lobe with antibodies against the AmOA1 receptor from rabbit (G1, magenta, Ranti-AmOA1) and goat (G2, green Ganti-AmOA1), reveal staining in the same cell bodies (lateral cluster, LG) and the same processes in the glomerular neuropil as shown by white in the merged image (G3). H: Control of immunostaining in agarose sections in the wild type (WT) Drosophila brain. The Ranti-AmOA1 antibodies recognized the OAMB receptor in the mushroom body (α/β' lobes and spur region of the pedunculus). A high level of staining is also observed in the anterior superiormedial protocerebrum (asmpr). I: In the oamb96 mutant, staining in the mushroom body is not present. J: In the antennal lobe (ant lobe) of a wild type fly, the Ranti-AmOA1 antibody recognizes cell bodies surrounding the antennal lobe neuropil and processes in the glomerular neuropil. K: In an oamb96 mutant fly, specific staining in the glomerular neuropil and cells is absent. Scale bars: 25 μm.
(23.11 MB TIF)
