# Supplementary figures and images for: Distribution of the Octopamine Receptor AmOA1 in the Honey Bee Brain
Source: PLoS One. 2011 Jan 18;6(1):e14536. doi: 10.1371/journal.pone.0014536 (PMC3022584; doi:10.1371/journal.pone.0014536)

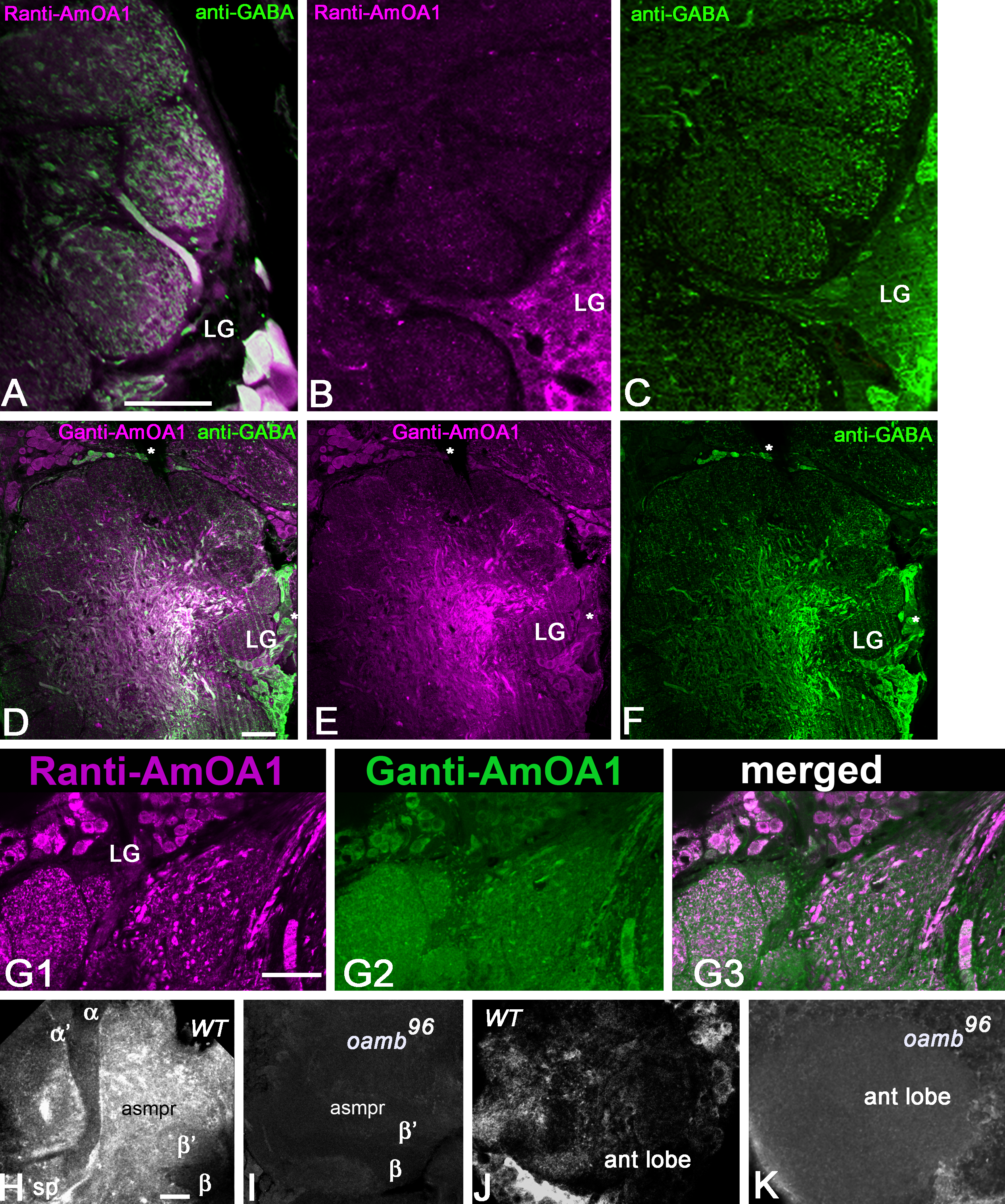

Supplement: Figure S1 — Characterization of anti-AmOA1 antibodies and double staining controls. A: Double immunostaining of the bee antennal lobe with anti-GABA and Ranti-AmOA1 antibodies B, C: Two consecutive sections of the honey bee antennal lobe for control of sequential staining with Ranti-AmOA1 (B) and GABA (C) antisera where the GABA or AmOA1 antibodies were omitted. D-F: Double immunofluorescence staining in the bee antennal lobe with anti-GABA and anti-AmOA1 antibodies from goat (Ganti-AmOA1). The staining reveals that most GABAergic neurons in the lateral group (LG) are positive for AmOA1. Neurons that have low intensity staining with AmOA1 but a high level of staining with anti-GABA are shown by an asterisk. G1-G3: Comparisons of immunostaining of the anti-AmOA1 from rabbit (Ranti-AmOA1) with anti-AmOA1 from goat (Ganti-AmOA1) on the same section of the antennal lobe. Double fluorescence staining in the antennal lobe with antibodies against the AmOA1 receptor from rabbit (G1, magenta, Ranti-AmOA1) and goat (G2, green Ganti-AmOA1), reveal staining in the same cell bodies (lateral cluster, LG) and the same processes in the glomerular neuropil as shown by white in the merged image (G3). H: Control of immunostaining in agarose sections in the wild type (WT) Drosophila brain. The Ranti-AmOA1 antibodies recognized the OAMB receptor in the mushroom body (α/β' lobes and spur region of the pedunculus). A high level of staining is also observed in the anterior superiormedial protocerebrum (asmpr). I: In the oamb96 mutant, staining in the mushroom body is not present. J: In the antennal lobe (ant lobe) of a wild type fly, the Ranti-AmOA1 antibody recognizes cell bodies surrounding the antennal lobe neuropil and processes in the glomerular neuropil. K: In an oamb96 mutant fly, specific staining in the glomerular neuropil and cells is absent. Scale bars: 25 μm. (23.11 MB TIF) [file pone.0014536.s001.tif]
